# The Potential Renal Acid Load of Edible Marine and Land Snails

**DOI:** 10.1038/s41538-026-00878-5

**Published:** 2026-05-07

**Authors:** Maximilian Andreas Storz

**Affiliations:** Department of Internal Medicine II, Centre for Complementary Medicine, Medical Center – University of Freiburg, Faculty of Medicine, University of Freiburg, Freiburg, Germany

**Keywords:** Biochemistry, Environmental sciences, Zoology

## Abstract

Edible snails, such as the giant West African land snail (*Archachatina marginata*), are an important protein source in many African countries. The potential renal acid load (PRAL), an estimate of the capability of a food to alter net endogenous acid or base production, of snail meat has not been examined so far. A quantification study of the PRAL of *n* = 40 snail species showed that snail meat fared worse (e.g., more acidic) than high-protein plant foods but better than most other high-protein animal food sources (e.g., less acidifying than pork or poultry), which may be attributable to the high calcium and low phosphorus content of snail meat.

Diet composition is an important determinant of the acid-base balance in humans^1^. Foods high in sulfur-containing amino acids and phosphate may increase the potential renal acid load (PRAL)—an estimate of the acid or base production in the human body subsequent to the intake of a certain food^1,2^. Most plant foods are rich in base precursors and thus exert alkalizing properties in the human body after ingestion^2^. Animal foods, such as dairy, cheese, and meat products, usually exert acidifying properties^3^.

A diet deficient in plant foods may result in a high PRAL^2,3^, which has been associated with acid stress and adverse health outcomes such as kidney damage, cardiovascular disease, and systemic inflammation^4–7^.

PRAL quantification is thus of paramount importance for individuals who wish to maintain an alkaline diet^2^. PRAL lists, which include the PRAL in mEq (milliequivalents) per 100 g of a certain food, can be used for dietary guidance^1^. However, novel and uncommon foods in Western countries are usually not included in the commonly used PRAL list, which dates back to the mid-1990s^1^. This applies, for example, to marine algae^8^, non-dairy plant milks^9^, plant-based meat analogs^10^, and edible insects^11^.

Edible marine and land snails have, *to the best of our knowledge,* not been analyzed in detail from an acid-base perspective on foods. Snails are traditionally consumed in many countries all over the world and are known for their high protein and calcium content^12–28^. Compared to conventional meats (e.g., beef or poultry), snail meat is also lower in phosphorus. These factors are of interest from a PRAL-centric perspective on foods and warrant further investigation. We sought to address this “PRAL-gap” in the literature and estimated the PRAL for some frequently consumed land and marine snails based on previous publications.

## Findings

Data for *n* = 40 edible snails were extracted from the literature^12–28^. The extracted data stems largely from Africa (e.g., Nigeria (*n* = 19) and Ghana (*n* = 3)) but also from India (*n* = 4) and other parts of the world. Table 1 summarizes the nutrient content per 100 g edible portion of the examined snails based on fresh matter analyses. In a similar style, Table 2 summarizes nutrient content per 100 g of food based on dry matter analyses.

**Table 1:** Nutrient content and PRAL values of edible snails based on 100 g fresh mass, based on data from refs. ^12–20^

Author(s)	Year	Country	Snail	Protein (g/100 g)	Potassium (mg/100 g)	Calcium (mg/100 g)	Magnesium (mg/100 g)	Phosphorus (mg/100 g)	PRAL (mEq/100 g)
Ab Lah et al.^12^	2016	Australia	*Lunella torquata*	18.03	305	239	69	153	3.19
Ab Lah et al.^12^	2016	Australia	*Lunella undulata*	18.49	333	44	65	164	5.87
Ab Lah et al.^12^	2016	Australia	*Turbo militaris*	16.19	273	61	77	122	3.92
Adeyeye^13^	1996	Nigeria	*Archachatina marginata*	20.76	44.18	212.38	46.33	0.64	5.30
Adeyeye^13^	1996	Nigeria	*Archatina sp*.	14.52	49.88	188.96	37.79	215.48	10.60
Adeyeye^13^	1996	Nigeria	*Limicolaria sp*.	17.51	78.82	22.24	18.91	183.35	12.93
Caetano et al.^14^	2021	Morocco	*Theba pisana*	11.40	77.5	364	66.6	140	2.67
Caetano et al.^14^	2021	Morocco	*Otala lactea*	9.60	73	293	58.7	128	2.57
Çağıltay et al.^15^	2011	Turkey	Helix aspersa*	12.87	105.4	135.7	17.05	96.72	5.46
Debnath et al.^16^	2016	India	*Brotia costula*	12.91	161.87	236.07	17.93	116.87	3.72
Debnath et al.^16^	2016	India	Bellamya bengalensis**	13.14	143.8	227.13	21.43	93.53	3.37
Debnath et al.^16^	2016	India	Bellamya dissimilis***	11.18	118.2	142.5	13.37	55.39	2.85
Debnath et al.^16^	2016	India	*Pila globosa*	15.59	182.28	312.5	18.5	121.17	3.74
Fagbuaro et al.^17^	2006	Nigeria	*Archachatina marginata (ovum) Pfeiffer*	20.56	192.78	201.08	45.59	123.43	6.79
Fagbuaro et al.^17^	2006	Nigeria	*Archachatina marginata (suturalis) Philippi*	20.34	209.95	207.53	45.34	123.23	6.24
Fagbuaro et al.^17^	2006	Nigeria	*Achatina achatina*	19.27	193.74	204.63	46.15	131.38	6.37
Fagbuaro et al.^17^	2006	Nigeria	*Limicolaria spp*.	18.66	197.57	208.75	45.99	153.89	6.78
Kehinde^18^	2019	Nigeria	*Archachatina marginata (adult)*	17.22	92.34	42.19	59.23	295.64	15.35
Kehinde^18^	2019	Nigeria	*Archachatina marginata (grower)*	16.30	77.55	31.64	60.04	286.65	14.99
Kehinde^18^	2019	Nigeria	*Archachatina marginata (snailet)*	15.83	69.24	26.46	238.8	274.5	9.91
Mason et al.^19^	2014	New Zealand	*Cookia sulcata (small)*	17.50	300	140	330	110	−4.06
Mason et al.^19^	2014	New Zealand	*Cookia sulcata (large)*	17.60	280	70	310	90	−2.90
Özogul et al.^20^	2005	Turkey	*Helix pomatia*	16.35	82.17	726.25	54.05	104.52	−0.69

*Taxonomic revision: now called Cornu aspersum.

**Now called Filopaludina bengalensis.

***Now called Idiopoma dissimilis.

**Table 2:** Nutrient content and PRAL values of edible snails based on 100 g dry mass, based on data from refs. ^21–28^

Author(s)	Year	Country	Snail	Protein (g/100 g)	Potassium (mg/100 g)	Calcium (mg/100 g)	Magnesium (mg/100 g)	Phosphorus (mg/100 g)	PRAL (mEq/100 g)
Akinnusi et al.^21^	2018	Nigeria	*Archachatina marginata*	22.75	193.4	201.1	42.6	121.6	7.86
Akinnusi et al.^21^	2018	Nigeria	*Achatina achatina*	11.92	196.7	204.6	43.8	134.3	2.88
Eneji et al.^22^	2008	Nigeria	*Archachatina marginata ovum*	84.43	60.79	180.71	29.2	59.16	39.17
Eneji et al.^22^	2008	Nigeria	*Archachatina marginata suturalis*	80.95	70	179.09	28.52	51.49	37.03
Eneji et al.^22^	2008	Nigeria	*Limicolaria species*	71.75	64.52	172.79	27.33	59.79	33.06
Eneji et al.^22^	2008	Nigeria	*Lanistes varicus*	70.00	69.39	152.7	30.56	62.52	32.38
Eneji et al.^22^	2008	Nigeria	*Nucella lapillus*	82.25	72.4	181.49	31.73	60.19	37.82
Envin et al.^23^	2018	République de Côte d’Ivoire	*Limicolaria flammea*	46.65	132.45	165.45	13.57	336.53	30.02
Essé et al.^24^	2024	République de Côte d’Ivoire	*Achatina achatina*	56.70	121	143	5.65	116	27.53
Gosh et al.^25^	2018	Republic of Korea	*Pomacea canaliculata*	48.50	364.4	5162.2	56.9	550.4	-32.11
Gosh et al.^25^	2025	Republic of Korea	*Acusta despecta*	70.90	280.8	1282.6	327.3	1251.2	49.96
Gosh et al.^25^	2025	Republic of Korea	Achatina fulica *	44.20	203.1	2008.3	343	699.2	8.24
Nkansah et al.^26^	2021	Ghana	*Archachatina marginata*	85.12	111.43	701.79	308.7	268.53	32.15
Nkansah et al.^26^	2021	Ghana	*Achatina achatina*	71.66	114.65	656.9	304.62	241.9	25.20
Nkansah et al.^26^	2021	Ghana	Achatina fulica *	62.56	111.02	402.29	301.2	61.29	17.53
Ogbuagu^27^	2011	Nigeria	*Achatina achatina*	68.48	48.53	66.8	28	15.99	31.53
Udo et al.^28^	1994	Nigeria	*Limicolaria aurora*	51.40	533	401	771	636	12.27

*Taxonomic revision: now called *Lissachatina fulica (Bowdich, 1822).*

Fresh mass analyses revealed an average protein content of 16.17 ± 3.12 g/100 g and a median calcium content of 201.08 (175.07) mg/100 g, respectively. The median potassium and phosphorus contents were 143.8 (132.4) and 123.43 (59.48) mg/100 g, respectively. As for the dry mass analyses, an average protein content of 60.60 ± 21.02 g/100 g and a median calcium content of 201.1 (484.11) mg/100 g were found. The median potassium and phosphorus contents were 114.65 (126.7) and 121.6 (276.34) mg/100 g, respectively.

**Fig. 1:**
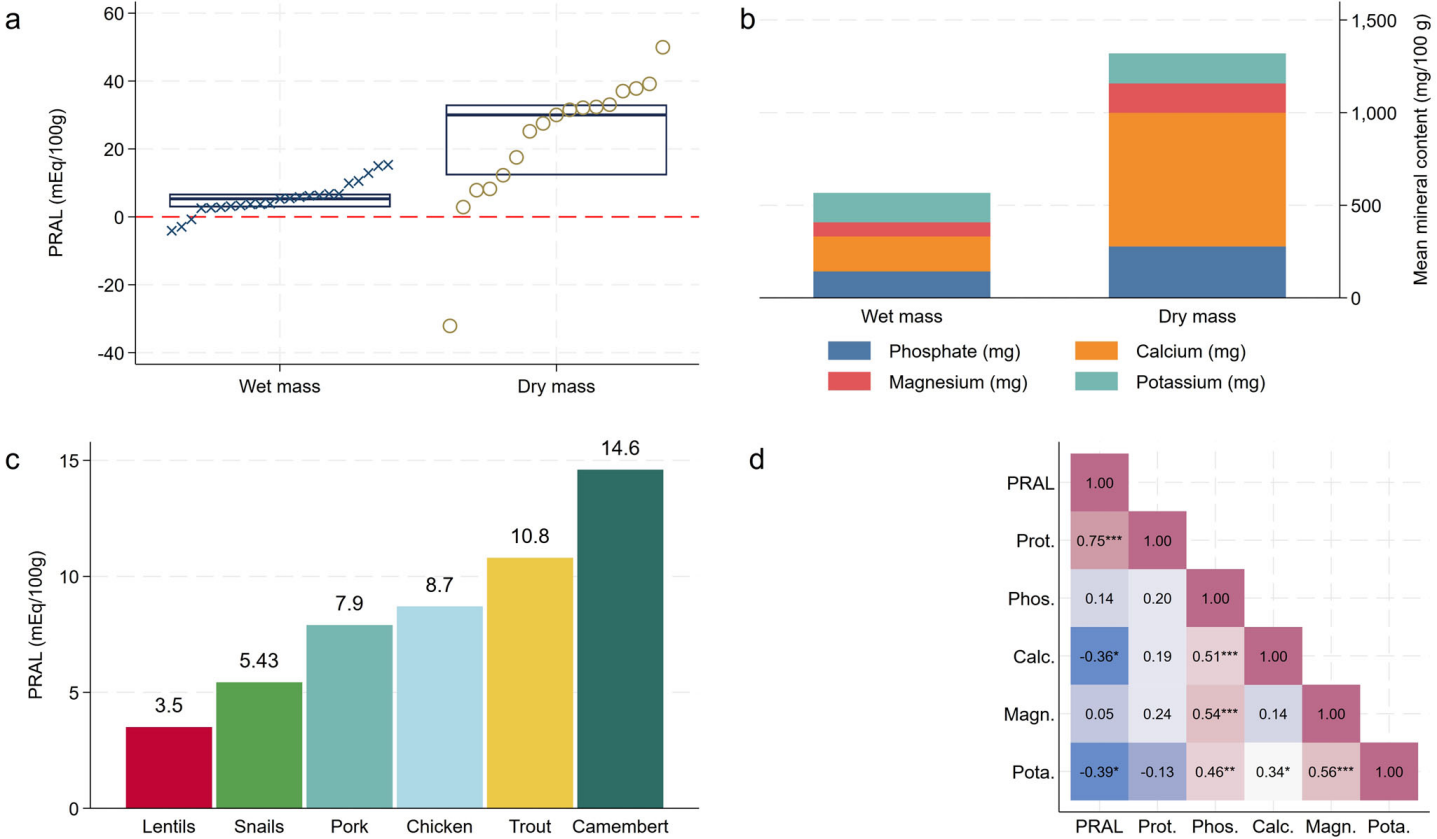
PRAL of edible marine and land snails. PRAL value (in mEq/100 g) of edible marine and land snails depending on dry mass or wet mass (panel **a**). Panel **b** displays the nutrient content per 100 g edible food portion. Panel c contrasts the PRAL of snails to other high-protein foods, including lentils, pork, and chicken. Panel **d** displays a correlation matrix for all PRAL-relevant nutrients in snail meat. In the correlation matrix, “***” indicates a *p*-value < 0.001; “**” indicates a *p*-value < 0.01, and “*” indicates a *p*-value < 0.1.

The estimated PRAL values ranged from −4.06 to +15.35 mEq/100 g in fresh mass analyses and from −32.11 to +49.96 mEq/100 g in dry mass analyses. Focusing on fresh mass results, *Archachatina marginata* (the giant West African land snail) and *Limicolaria sp*. showed the highest PRAL values (Table 1). Dry mass analyses suggested the highest PRAL for *Acusta despecta* and the lowest for *Pomacea canaliculata*, potentially due to the extremely high calcium content of the latter (Table 1). Mean/median PRAL values were 5.43 ± 4.93 for fresh mass and 30.02 (20.79) mEq/100 g for dry mass, respectively. Results are visualized in Fig. 1. Only 10% of the examined snails had a PRAL < 0 mEq, whereas PRAL values for the remaining 90% suggested moderate to strong acidifying effects. Panel c displays a bar chart with average PRAL values for some commonly consumed high-protein foods. While the average PRAL of snails was lower in comparison to most other meats, it was higher when compared to lentils (5.43 mEq/100 g vs 3.5 mEq/100 g), which are at the same time characterized by a higher total protein content (24.3 g/100 g vs. 16.6 g/100 g). Figure 1, panel d displays a correlation matrix of the examined nutrients and the PRAL. The positive associations between calcium and phosphorus and between magnesium and phosphorus are particularly noteworthy in this context. Both associations suggest that snails with a higher phosphorus content are also richer in base precursors, which reduces snails’ overall acidifying potential.

## Summary and discussion

The present study explored the PRAL of snail meat, which is an important food and protein source for many inhabitants in sub-Saharan Africa and Asia^29,30^. Snails are known for their high protein content and are rich in essential amino acids, calcium, and magnesium^30^. In low-resource settings, snails may help to address malnutrition and stunting^31,32^. In the context of helicicultural facilities^33^, however, a debate about the safety profiles of snail meats has emerged^31^. Some authors suggested that edible land snails may contain microplastics, nanoparticles, and other biological hazards^31^. Against the background of this debate, we deemed it essential to explore the PRAL of edible land and marine snails. High-PRAL diets may result in acid stress and adverse health outcomes^5,11^, while the PRAL of most snail meats is virtually unknown.

Our results suggest that snails exert acidifying properties. However, the PRAL of snails appears lower in comparison to most other animal-based high-protein foods, including pork and chicken. In comparison to most high-protein plant foods, such as beans and lentils, the average PRAL of snails was slightly higher (Fig.1c). The high calcium content of snails could play a pivotal role in this context, although absorption rates and bioavailability are, to the best of our knowledge, unknown for snails. One important non-negligible aspect is the low phosphorus content of snails, which may result in lower PRAL values as compared to many other high protein sources^1^. Our analysis revealed a median phosphate content of 123.43 (59.48) mg/100 g fresh mass. On the other hand, beef and pork contain on average 185 mg of phosphate per 100 g of meat^34^. Chicken breast contains even higher amounts of phosphate (213 mg/100 g). These differences, in conjunction with snails’ high calcium content, could be important factors explaining the less acidifying potential of snails in comparison to other meats.

Our results may be helpful to complete and update the 1995-PRAL list and suggest an average PRAL value of 5.4 mEq/100 g of fresh snail meat. The herein presented data might be of high importance in the context of diets low in base precursors/plants and help to quantify the average PRAL of snails. Limitations include the potentially incomplete search strategy, the consideration of only *n* = 40 food items, and potential heterogeneity in the nutrient content assessments in the original studies. It is also important to note that taxonomic revisions of various snail genera occurred over the past years^35^. This applies, for example, to *Cornu aspersum*, which was formerly known as *Helix aspersa Müller*^36^. For this brief communication, we decided to present names as provided in the original publications and added footnotes including taxonomic revisions (where applicable). The herein presented data largely stem from the African continent, and many newer publications could not be included because PRAL-relevant nutrients were not fully reported (e.g., Nnamonu et al. and Manet et al.^37,38^). Despite these potential limitations, we believe that this brief communication could serve as an important supplement to the 1995-PRAL list and offers preliminary insights into the PRAL of edible snails.

## Methods

This work is part of a larger series quantifying the PRAL of “novel” food items and follows previously explained methods^8–11^. In brief, nutrient profiles for edible land and marine snails were obtained through a thorough literature search, which covered publications in English deposited in PubMed, Scopus, and Google Scholar. The literature search strategy included a combination of search terms, including: snail, gastropod, nutrient content, nutrient composition, nutritional value, protein, and minerals. Additional publications were identified using review articles^31,32^. The search was not designed to reflect a systematic review. Only data from snails with a complete nutrient profile required for the PRAL estimation (see equation below) were extracted. Publications that did not contain all PRAL-relevant nutrients were discarded. The search was conducted by the author in December 2025.

The PRAL in mEq was estimated using the following nutrient intake-based formula per 100 g of food^1^:

$${\mathrm{PRALR}}\left(\frac{mEq}{100g}\right)=0.49\times protein\left(\frac{g}{100g}\right)+0.037\times phosphorus\left(\frac{mg}{100g}\right)-0.021\times potassium\left(\frac{mg}{100g}\right)\,\,\,\,\,\,\,\,\,\,\,\,\,\,\,\,\,\,\,\,\,\,\,\,\,\,\,\,\,\,-0.026\times magnesium\left(\frac{mg}{100g}\right)-0.013\times calcium\left(\frac{mg}{100g}\right)$$

Data was analyzed in Stata 18 statistical software (StataCorp, 2023; Stata Statistical Software: Release 18. College Station, TX: StataCorp LLC). The data distribution was analyzed via histograms and the Shapiro–Wilk normality test. Means (±SD) or medians (+interquartile range) were provided depending on the data distribution. The PRAL was separately estimated for fresh- and dry matter-based analyses. Mean PRAL values based on fresh matter analyses were contrasted to other selected high-protein foods with PRAL values obtained from Remer and Manz^1^. Pearson’s product-moment correlations were run to assess the relationship between PRAL-relevant nutrients in snails.

## Data Availability

All data generated or analyzed during this study are included in this published article.
